# CD14 Mediates Binding of High Doses of LPS but Is Dispensable for TNF-****α**** Production

**DOI:** 10.1155/2013/824919

**Published:** 2013-12-30

**Authors:** Kinga Borzęcka, Agnieszka Płóciennikowska, Hanna Björkelund, Andrzej Sobota, Katarzyna Kwiatkowska

**Affiliations:** ^1^Department of Cell Biology, Nencki Institute of Experimental Biology, 3 Pasteur Street, 02-093 Warsaw, Poland; ^2^Ridgeview Instruments AB, Skillsta 4, 740 20 Vänge, Sweden; ^3^Biomedical Radiation Sciences, Department of Radiology, Oncology and Radiation Sciences, Uppsala University, Dag Hammarskjölds väg 20, 751 85 Uppsala, Sweden

## Abstract

Activation of macrophages with lipopolysaccharide (LPS) involves a sequential engagement of serum LPS-binding protein (LBP), plasma membrane CD14, and TLR4/MD-2 signaling complex. We analyzed participation of CD14 in TNF-**α** production stimulated with 1–1000 ng/mL of smooth or rough LPS (sLPS or rLPS) and in sLPS binding to RAW264 and J744 cells. CD14 was indispensable for TNF-**α** generation induced by a low concentration, 1 ng/mL, of sLPS and rLPS. At higher doses of both LPS forms (100–1000 ng/mL), TNF-**α** release required CD14 to much lower extent. Among the two forms of LPS, rLPS-induced TNF-**α** production was less CD14-dependent and could proceed in the absence of serum as an LBP source. On the other hand, the involvement of CD14 was crucial for the binding of 1000 ng/mL of sLPS judging from an inhibitory effect of the anti-CD14 antibody. The binding of sLPS was also strongly inhibited by dextran sulfate, a competitive ligand of scavenger receptors (SR). In the presence of dextran sulfate, sLPS-induced production of TNF-**α** was upregulated about 1.6-fold. The data indicate that CD14 together with SR participates in the binding of high doses of sLPS. However, CD14 contribution to TNF-**α** production induced by high concentrations of sLPS and rLPS can be limited.

## 1. Introduction

Mechanisms of the innate immunity assure a rapid response directed against microbes which have successfully overcome physical barriers protecting the body. These reactions are triggered upon recognition of evolutionarily conserved constituents of microorganisms named pathogen-associated molecular patterns (PAMPs) by distinct cellular receptors among which Toll-like receptors (TLR) are of great importance [1]. The prototypical PAMP is lipopolysaccharide (LPS, endotoxin), a major constituent of the outer membrane of Gram-negative bacteria. LPS activates TLR4 of leukocytes and initiates signalling cascades leading to production of proinflammatory mediators exemplified by tumor necrosis factor-*α* (TNF-*α*), chemokines like MIP-2 and RANTES, and type I interferons [2, 3]. The presence of high LPS concentrations in the blood and the following exaggerated production of TNF-*α* and other pro-inflammatory mediators can lead to a systemic inflammatory reaction, termed sepsis [4, 5].

LPS molecules consist of three components: the polysaccharide chain named the O-antigen, the core oligosaccharide and lipid A with the latter determining the proinflammatory activity of endotoxin. The greatest variability in LPS structures is observed within the O-specific chain and concerns the chemical nature and the number of sugar residues assembling the polysaccharide, as well as the position and stereochemistry of the *O*-glycosidic linkages [6, 7]. In certain species or mutants of Gram-negative bacteria, or in distinct growth conditions, the O-specific chain may be absent giving rise to a so-called rLPS (from a “rough” phenotype of bacterial colonies) in contrast to the typical phenotype of “smooth” colonies synthesizing sLPS with the O-antigen. The lack of the O-specific chain modulates the process of LPS recognition by cells of the immune system which can lead eventually to differences in the magnitude of the cytokine production, as it was found for LPS originating from *Salmonella sp.*, *Brucella sp.*, and *Escherichia coli* [8–10].

An optimal response of macrophages to LPS requires a cooperation of a number of extracellular and plasma membrane proteins, including serum LPS-binding protein (LBP) which monomerizes LPS and transfers LPS molecules to the plasma membrane-anchored CD14 [11]. CD14 is 56 kDa protein which forms homodimers and binds lipid portion of LPS in its NH_2_-terminal hydrophobic pocket [12, 13]. The protein is incorporated in the outer leaflet of the plasma membrane via a glycosylphosphatidylinositol anchor and contains no transmembrane or cytoplasmic domains. It was the reason why CD14 together with LBP was assumed to play merely a role of sensors efficiently capturing LPS molecules and transferring them to a signalling complex composed of MD-2 protein associated with TLR4. Dimerization of TLR4/MD-2 complexes induced upon LPS binding triggers two signalling pathways depending on the association of TLR4 with either MyD88/TIRAP or TRIF/TRAM adaptor proteins, respectively [14–17]. Recent studies indicate that CD14 is important for the initiation of proinflammatory signalling triggered by sLPS rather than rLPS [10]. However, CD14 may fulfil also other functions in the process of cell stimulation than simple LPS recognition. In macrophages isolated from mice with mutant CD14, the TRIF-dependent signalling pathway of TLR4 was nullified [9]. This disabled pathway was linked to CD14-dependent endocytosis of LPS-activated TLR4 [18]. CD14 participates also in LPS internalization in a pathway which leads to an intracellular detoxification of LPS. This LPS uptake is attributed mainly to the activity of scavenger receptors (SR) and cooperation of SR with CD14 was indicated [19–22].

On the other hand, CD14 is not the only one coreceptor of TLR4 in LPS-stimulated cells. Measurements of the resonance energy transfer between fluorescently labelled membrane proteins in LPS-stimulated monocytes revealed that activated TLR4 coclustered with CD14 and also with heat-shock proteins 70 and 90, CD55, CD11/CD18, and chemokine receptor 4 (CXCR4) [23, 24]. These proteins can participate in LPS-induced production of TNF-*α* by functioning as LPS-binding molecules similarly to CD14; however, signalling properties of CXCR4 were also indicated [25, 26].

The complexity of the TLR4-accompanying plasma membrane receptors potentially involved in LPS recognition prompted us to analyze the participation of CD14 in TNF-*α* production stimulated by sLPS and rLPS of *E. coli* and in sLPS binding. We found that CD14 moderately affected TNF-*α* production induced by high doses of sLPS and rLPS. On the other hand, CD14 together with SR participated in the binding of high doses of sLPS. The data suggest that the involvement of CD14 is important for recognition and binding of sLPS. However, CD14 contribution to TNF-*α* production induced by high doses of sLPS and rLPS can be limited.

## 2. Materials and Methods

### 2.1. Cell Culture and Stimulation

RAW264 and J774A.1 cells were cultured in DMEM medium supplemented with 10% fetal bovine serum (FBS) at 5% CO_2_. Cells were stimulated with ultrapure smooth LPS (sLPS) from *E. coli* 0111:B4 (List Biological Laboratories) or rough LPS (rLPS) from *E. coli*, serotype 515, Re mutant (Enzo), or *S*-[2,3-bis(palmitoyloxy)-propyl]-(*R*)-cysteinyl-(lysyl)3-lysine (Pam_2_CSK_4_), or *N*-palmitoyl-*S*-[2,3-bis(palmitoyloxy)-propyl]-(*R*)-cysteinyl-(lysyl)3-lysine (Pam_3_CSK_4_), or polyinosinic-polycytidylic acid (poly(I:C)) (all from InvivoGen). When indicated, cells were exposed to sLPS labeled with Alexa Fluor 488 hydrazide (Molecular Probes) or hydrazide-LC-biotin (Thermo Scientific) according to [27]. Concentration of LPS after labeling was estimated using Pierce LAL Chromogenic Endotoxin Quantitation Kit (Thermo Scientific). To determine the AF488 content in LPS-AF488 samples, their absorbance at 492 nm was measured. A 5 : 1 labeling ratio of AF488-to-LPS was obtained. Labeling of LPS with biotin was confirmed by dot-blot analysis of the binding of streptavidin-peroxidase (Sigma) to LPS-biotin applied onto a nitrocellulose in a 5–50 ng/mL range. Labeling of sLPS either with AF488 or biotin did not diminish endotoxin activity, as indicated by its ability to induce TNF-*α* production in comparison to sLPS prior to the labeling.

### 2.2. Silencing of TLR4 Gene

RAW264 cells (1.5 × 10^5^) were suspended in 1 mL of RPMI medium supplemented with 5% FBS, mixed with 1 mL of serum-free RPMI containing 20 *μ*L of TrueFect-Lipo (United BioSystems) and 200 pmol of either TLR4 small interfering RNA (siRNA) or control scrambled siRNA (both Ambion), and seeded onto 35 mm culture plates. After 6 h, the medium was changed to DMEM/10% FBS, and cells were cultured for 24 h, plated in 96-well plates (0.5 × 10^5^/well), and cultured for 20 h for further experiments.

### 2.3. TNF-*α*, RANTES, and MIP-2 Assays

Cells (0.5 × 10^5^/well in 96-well plates) were stimulated with 1–1000 ng/mL of sLPS or rLPS, or 10–100 *μ*g/mL of Pam_3_CSK_4_, or 10–100 *μ*g/mL of Pam_2_CSK_4_, or 5–20 *μ*g/mL of poly(I:C) at 37°C in the presence or absence of 10% FBS. When indicated, prior to stimulation cells were incubated for 30 min at 37°C with 10 *μ*g/mL of function blocking anti-CD14 rat IgG2b, clone 4C1, or 10 *μ*g/mL isotype-matched control rat IgG2b (both Becton Dickinson), or 50 *μ*g/mL chondroitin sulfate A from bovine trachea, or 50 *μ*g/mL dextran sulfate from *Leuconostoc* ssp. (MW 500,000) (both Sigma) or combination of the antibodies and chondroitin sulfate or dextran sulfate. Subsequent stimulation of cells with either LPS or other above-mentioned ligands was conducted in the presence of appropriate drugs and antibodies. Concentrations of sulfates and antibodies were reduced by half after LPS addition. Levels of TNF-*α* and MIP-2 in culture supernatants were determined after 4 h while RANTES after 6 h of stimulation, with an application of murine ELISA kits (R&D Systems, Biolegends, Peprotech). The product absorbance was measured using a Sunrise plate reader (Tecan Group).

### 2.4. LigandTracer Binding Assay

J774 cells (1 × 10^6^ in 3 mL of DMEM/10% FBS) were seeded in a local area of tilted 8.7 cm cell dish according to [28] and cultured at 5% CO_2_, 37°C. After 6 h, the dish was supplemented with 7 mL of DMEM/10% FBS and cells were grown overnight. Prior to experiments, cells were washed once with DMEM/10% FBS supplemented with 20 mM Hepes, pH 7.4, layered with 2 mL of the medium containing the mixture of 10 *μ*g/mL anti-CD14 rat IgG2b (clone 4C1) and 50 *μ*g/mL dextran sulfate or 50 *μ*g/mL chondroitin sulfate A and incubated for 15 min at 37°C. The dish was placed on a tilted, rotating support of the instrument LigandTracer Green (Ridgeview Instruments AB, Uppsala, Sweden) in an incubator at 37°C/5% CO_2_ for the baseline setting. After 30 min, 40 *μ*L of sLPS-AF488 was added to the medium to final concentration of 3 *μ*g/mL of LPS and real-time LPS-cell association was monitored for 1 h. In this time, repeated measurements of the fluorescence from dish areas covered with and devoid of cells were performed generating an output signal defined as a difference between the fluorescence of the cell-containing area and the fluorescence of the surrounding reference area. After removal of the medium and washing, the dish was filled with 2 mL of DMEM/10%FBS/20 mM Hepes, pH 7.4, with appropriate drugs, and the measurements were carried out for another 1 h as above mentioned to follow retention of LPS in cells. After adding 100 *μ*g/mL of concanavalin A-FITC to the medium, the measurements were continued for 30–40 min to assess the association of the lectin with cells.

### 2.5. Binding and Internalization of LPS-Biotin

Cells were plated at 4 × 10^4^/well in 96-well plates in DMEM/10% FBS. After 18 h, cells were incubated in the presence of 10 *μ*g/mL anti-CD14 antibody or 10 *μ*g/mL control rat IgG2b, or 50 *μ*g/mL chondroitin sulfate or 50 *μ*g/mL dextran sulfate or a combination of those compounds in DMEM/10% FBS (30 min, 37°C). Subsequently, the cultures were supplemented with sLPS-biotin at 1 *μ*g/mL reducing concentration of antibodies and sulfates by half. After 1 h (37°C), cell were washed twice with PD buffer (125 mM NaCl, 4 mM KCl, 10 mM NaHCO_3_, 1 mM KH_2_PO_4_, 10 mM glucose, 20 mM Hepes, pH 7.4) and to facilitate uptake of sLPS-biotin they were incubated in DMEM/10% FBS for another 1 h in the presence of appropriate drugs or antibodies. After final wash with PD buffer, cells were exposed to 150 *μ*L of a hypotonic solution of 2 mM EGTA, 2 mM EDTA, 20 mM Hepes, pH 7.4 (10 min, 4°C) and sonicated on ice for 5 min at 0.25 cycle, amplitude 25% using an UP200S Hielscher sonifier (Germany). The homogenates were transferred into eppendorf tubes and centrifuged (10 min, 10 000 g, 4°C); supernatants were diluted twice with TBS buffer and applied in 100 *μ*L quantities onto nitrocellulose membranes. After blocking with 3% bovine serum albumin in TBS buffer containing 0.05% Tween 20, blots were incubated with streptavidin-peroxidase and immunoreactive dots were visualized by chemiluminescence, using SuperSignal West Pico substrate (Pierce).

To assess the binding of sLPS-biotin but to prevent its internalization, cells were preincubated for 30 min at 37°C with 0.05% NaN_3_ prior to adding 5–15 *μ*g/mL of the anti-CD14 antibody, or 10 *μ*g/mL of control rat IgG2b or 50 *μ*g/mL of chondroitin sulfate or 50 *μ*g/mL of dextran sulfate or the mixture of 5 *μ*g/mL anti-CD14 antibody and 50 *μ*g/mL dextran sulfate. After 30 min (37°C), cultures were supplemented with 1 *μ*g/mL of sLPS-biotin for 1 h (37°C) in the presence of 0.05% NaN_3_. Cells were washed twice with PD buffer and processed for dot-blot analysis as mentioned above. Blots were analyzed densitometrically using the ImageJ software. For normalization, dot intensity values found in nonstimulated cells and reflecting a nonspecific binding of streptavidin-peroxidase to cell homogenates were subtracted from those found in LPS-treated cells. The data are expressed in relation to LPS level found in cells exposed to cIgG and arbitrarily equalized to 100.

### 2.6. Immunoblotting

Proteins of whole cell lysates were separated by 10% SDS-PAGE, transferred onto nitrocellulose, and probed with rabbit anti-TLR4 (Santa Cruz Biotechnology) and mouse anti-actin antibodies (BD Biosciences) followed by goat anti-rabbit or anti-mouse IgG conjugated with peroxidase. Immunoreactive bands were visualized and analyzed densitometrically as above to assess TLR4 level normalized against actin content in samples.

### 2.7. Data Analysis

The significance of differences between groups was calculated using Student's *t*-test. *P*  values ≤ 0.05 were considered to be statistically significant.

## 3. Results

### 3.1. CD14 Moderately Affects TNF-*α* Production Triggered by High Doses of LPS

**Figure 1:**
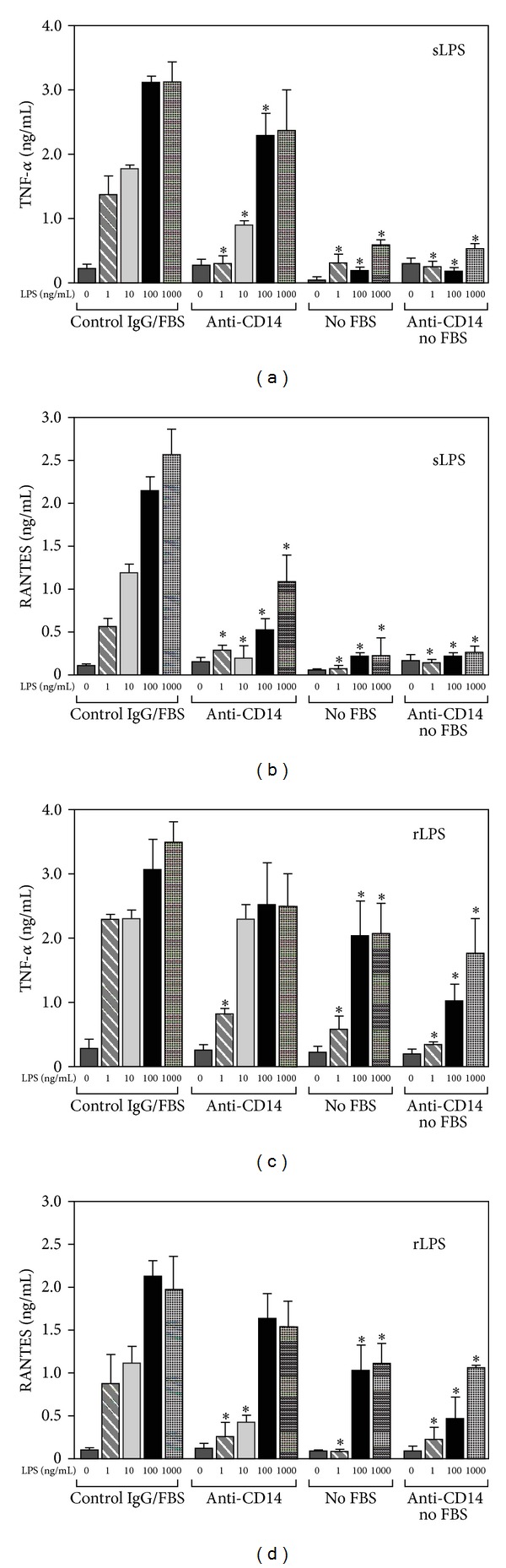
Interference with LPS/CD14 interaction moderately affects TNF-*α* production induced by high concentrations of sLPS or rLPS. RAW264 cells were pretreated with 10 *μ*g/mL of the anti-CD14 antibody, clone 4C1, or isotype-matched control rat IgG2b (30 min, 37°C) and subsequently stimulated with 1–1000 ng/mL sLPS (a, b) or 1–1000 ng/mL rLPS (c, d) in the presence or absence of 10% FBS, as indicated. Concentrations of TNF-*α* (a, c) and RANTES (b, d) were measured by ELISA tests in supernatants of the cells. Data shown are mean ± SEM form three or four experiments each run in triplicate. *Significantly different from cells stimulated with a corresponding LPS concentration in the presence of control IgG and FBS.

To asses the involvement of CD14 in LPS-induced signaling, we examined effects of neutralizing of CD14 on the production of TNF-*α* and RANTES. TNF-*α* is synthesized in MyD88-dependent while RANTES in TRIF-dependent signaling pathways of TLR4 [3]. RAW264 cells were stimulated with 1–1000 ng/mL of sLPS or rLPS of *E. coli* in the presence of 4C1 antibody which excluded LPS binding to CD14 [29]. We found that this blocking of function of CD14 nearly abolished TNF-*α* and RANTES production induced by 1 ng/mL of sLPS. In cells stimulated with 10 ng/mL of sLPS the antibody significantly, by 51% and 88%, reduced production of TNF-*α* and RANTES, respectively (Figures 1(a) and 1(b)). In contrast, there was a clear difference in the CD14 involvement in TNF-*α* and RANTES generation induced by higher, 100 and 1000 ng/mL, doses of sLPS. In these conditions, the blocking of the LPS/CD14 interaction by the 4C1 antibody inhibited the production of TNF-*α* by 25–27% only (Figure 1(a)) while RANTES generation was reduced by 58–76% (Figure 1(b)). For comparison, when stimulation of cells was conducted in a medium devoid of FBS as an LBP source, production of both TNF-*α* and RANTES induced by 1–1000 ng/mL sLPS was greatly inhibited approaching the level found in cells prior to the stimulation. When cells were deprived of FBS and additionally exposed to the anti-CD14 function blocking antibody, the cytokine release remained very low (Figures 1(a) and 1(b)).

As signaling properties of LPS can be modulated by the O-antigen polysaccharide chain [9, 10], we next examined the involvement of CD14 in rLPS-induced cytokine production. In cells stimulated with rLPS, TNF-*α* production was less dependent on CD14 than in cells exposed to sLPS. Neutralizing CD14 with the 4C1 antibody inhibited by 65% the production of TNF-*α* induced by 1 ng/mL rLPS. However, in cells stimulated with 10–1000 ng/mL of rLPS, the production of TNF-*α* was reduced by 5–30% only (Figure 1(c)). When used at higher concentrations, despite the presence of the CD14-neutralizing antibody, 100 or 1000 ng/mL of rLPS was able to induce as much as 77–82% of the RANTES production found in control cells (Figure 1(d)). In further contrast to sLPS, even in the absence of FBS, rLPS at 100 or 1000 ng/mL induced the production of TNF-*α* and RANTES which approached 47–66% of control levels (Figures 1(c) and 1(d)). The absence of FBS combined with the neutralizing of CD14 diminished strongly the production of TNF-*α* and RANTES induced by 1–100 ng/mL of rLPS. However, in these conditions rLPS at 1000 ng/mL was still able to induce synthesis of TNF-*α* and RANTES reaching about 51% and 58% of control values, respectively (Figures 1(c) and 1(d)). Taken together, the data indicate the following: (i) CD14 is dispensable for TNF-*α* production induced by higher (100–1000 ng/mL) concentrations of both rLPS and sLPS; (ii) rLPS, when used at higher doses, can bypass not only CD14 but also LBP involvement to activate TLR4-dependent release of TNF-*α* and RANTES.

**Figure 2:**
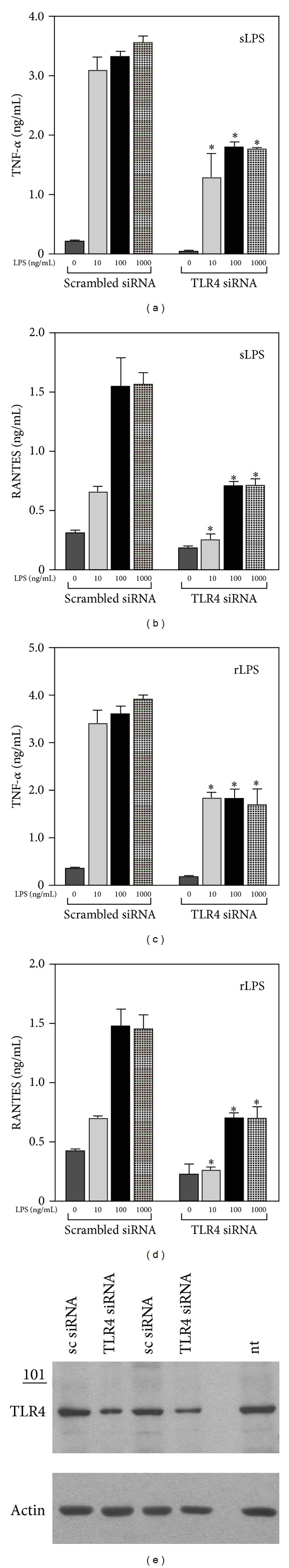
Silencing of TLR4 gene expression significantly downregulates production of TNF-*α* and RANTES in cells stimulated with sLPS or rLPS. RAW264 cells were transfected with TLR4-specific siRNA or scrambled siRNA and, after 50 h, stimulated with 10–1000 ng/mL sLPS (a, b) or 10–1000 ng/mL rLPS (c, d). Production of TNF-*α* (a, c) and RANTES (b, d) by the cells was estimated by ELISA tests. Data shown are mean ± SEM from three experiments each run in triplicate. *Significantly different from cells treated with scrambled siRNA and stimulated with a corresponding LPS concentration. (e) Immunoblotting analysis of TLR4 protein level versus actin level in cells transfected with TLR4-specific or scrambled (sc) siRNA. nt: not transfected cells. On the left, a molecular weight marker (prestained phosphorylase b, 101 kDa) is indicated. Data from two independent experiments are shown.

We then examined whether cytokine production induced by sLPS and rLPS in those lines of experiments was indeed attributed to TLR4 activation. The silencing of expression of TLR4-encoding gene in RAW264 cells suppressed TNF-*α* production induced by sLPS or rLPS by 47–59% regardless of LPS concentration (Figures 2(a) and 2(c)). Notably, downregulation of TLR4 inhibited RANTES release by 55–61% resembling the inhibitory effect exerted on TNF-*α* production (Figures 2(b) and 2(d)). This fairly even inhibition of cytokine production corresponded to 50–55% reduction of TLR4 level in cells transfected by specific siRNA in comparison to cells treated with scrambled siRNA (Figure 2(e)) pointing to TLR4 as mediating inflammatory responses to sLPS and rLPS in these studies.

**Figure 3:**
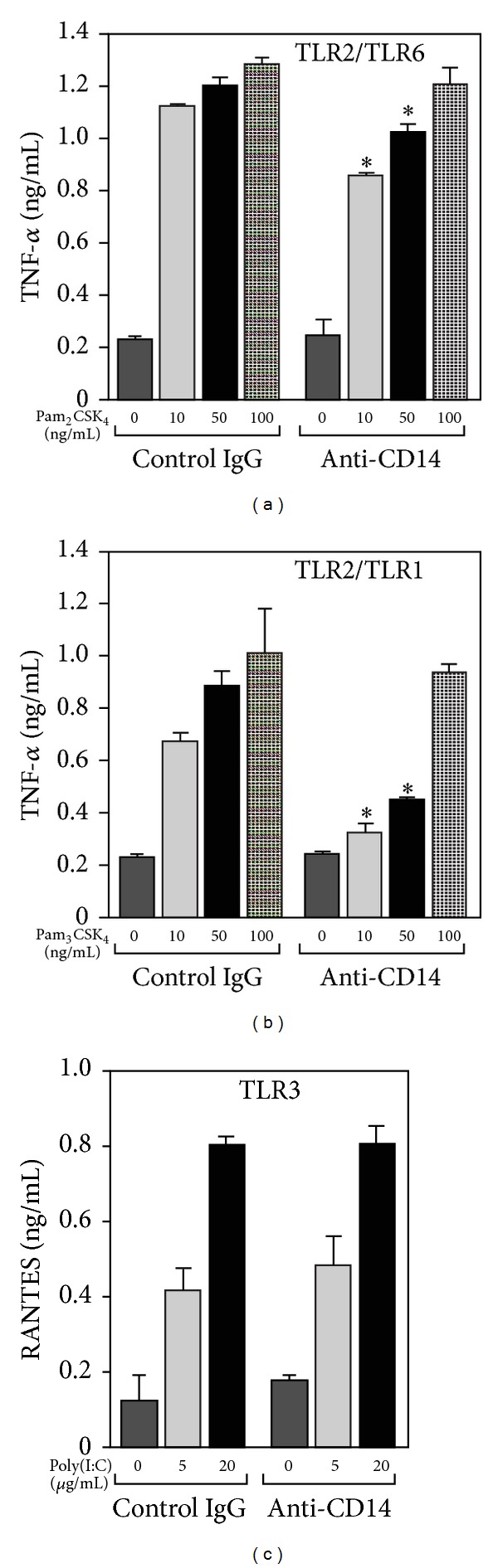
Effect of neutralizing CD14 on cytokine production during activation of TLR2/TLR6, TLR2/TLR1, and TLR3. RAW264 cells were pretreated with 10 *μ*g/mL of the anti-CD14 antibody or isotype-matched control rat IgG2b (30 min, 37°C) and exposed to indicated concentrations of Pam_2_CSK_4_ (a) or Pam_3_CSK_4_ (b) or poly(I:C) (c) in the presence of 10% FBS. Amounts of TNF-*α* (a, b) and RANTES (c) were measured by ELISA tests in supernatants of the cells. Data shown are mean ± SEM form two or three experiments each run in triplicate. *Significantly different from cells stimulated with a corresponding ligand concentration in the presence of control IgG.

We also analyzed whether the neutralizing of CD14 can affect production of TNF-*α* and RANTES induced by ligands of other TLRs in RAW264 cells. The anti-CD14 antibody induced partial inhibition of TNF-*α* production in a response to synthetic lipopeptides, Pam_2_CSK_4_ and Pam_3_CSK_4_, ligands of TL2/TLR6 and TLR2/TLR1, respectively. No inhibition of TNF-*α* release was found in cells exposed to 100 ng/mL of Pam_2_CSK_4_ or Pam_3_CSK_4_. However, at lower doses, 10–50 ng/mL, of the lipopeptides the production of TNF-*α* was suppressed by about 50% in cells stimulated with Pam_3_CSK_4_ and by 15–24% when Pam_2_CSK_4_ was used (Figures 3(a) and 3(b)). These results are in agreement with suggestions that CD14 serves as a sensor of amphipathic molecules [30–32], although data arguing against the involvement of mouse CD14 in TLR2/TLR1 signaling should be noted [9]. On the other hand, production of RANTES in cells stimulated with 5–20 *μ*g/mL of poly(I:C), a ligand of endosomal TLR3, was not changed by the anti-CD14 antibody (Figure 3(c)). The data indicate that CD14 is not involved in endocytosis of TLR3 ligands which can be delivered to the receptor by scavenger receptor A (SR-A) [33].

### 3.2. CD14 and Scavenger Receptors Participate in the Binding of High Doses of LPS

**Figure 4:**
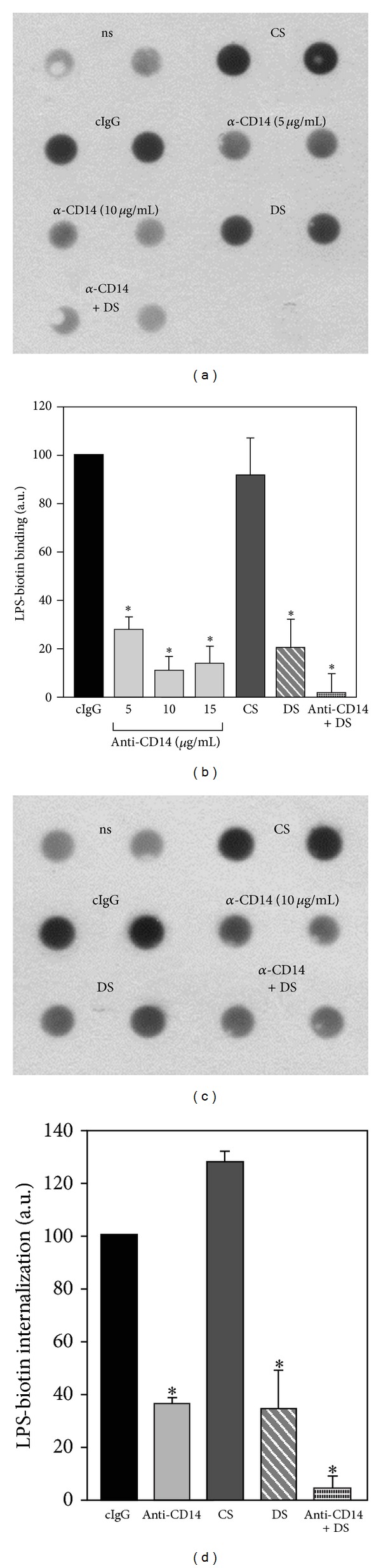
Binding of high doses of sLPS to the surface of RAW264 cells is mediated by CD14 and SR. (a, b) Binding of sLPS-biotin to the surface of RAW264 cells. Cells were preincubated with 0.05% NaN_3_ (30 min, 37°C) and either left untreated (ns) or exposed to 5 or 10 *μ*g/mL of the anti-CD14 antibody or 10 *μ*g/mL isotype-matched control IgG (cIgG) or 50 *μ*g/mL dextran sulfate (DS), or 50 *μ*g/mL chondroitin sulfate (CS), or the mixture of 5 *μ*g/mL anti-CD14 antibody and 50 *μ*g/mL dextran sulfate. After 30 min (37°C), cells were stimulated with 1 *μ*g/mL sLPS-biotin (1 h, 37°C) in the constant presence of 0.05% NaN_3_. The amount of sLPS-biotin bound to the cell surface was assessed by dot-blot analysis of cell homogenates using streptavidin-peroxidase (a). (c, d) Internalization of sLPS-biotin. Cells were preincubated with 10 *μ*g/mL anti-CD14 antibody or 10 *μ*g/mL isotype-matched control IgG or 50 *μ*g/mL dextran sulfate or 50 *μ*g/mL chondroitin sulfate or a mixture of the anti-CD14 and dextran sulfate for 30 min at 37°C. Subsequently, the samples were supplemented with 1 *μ*g/mL of sLPS-biotin for 1 h and washed and incubated for another 1 h at 37°C prior to homogenization and dot-blot analysis (c). (b, d) Quantification of cell surface-bound (b) and internalized (d) sLPS-biotin based on densitometric analysis of dot-blots. Data are mean ± SEM from three experiments. *Significantly different from cells exposed to control IgG.

The moderate effect exerted by the CD14 neutralizing antibody on TNF-*α* production induced by 100 or 1000 ng/mL of LPS can indicate that CD14 is not crucial for the binding of high doses of endotoxin. To test this assumption, we measured the binding and internalization of 1000 ng/mL of sLPS conjugated with biotin in RAW264 cells (Figure 4). To block sLPS internalization, the binding of the endotoxin was performed in the presence of 0.05% NaN_3_ (1 h), after which cells were homogenized and amounts of sLPS-biotin bound to the cell surface were measured by a dot-blot analysis. In these conditions, treatment of cells with 5–15 *μ*g/mL of the anti-CD14 antibody strongly inhibited the binding of 1000 ng/mL of sLPS. The amounts of bound sLPS were reduced by about 72% at 5 *μ*g/mL and by about 86–88% at 10–15 *μ*g/mL of the anti-CD14 antibody in comparison to cells treated with the isotype-matched control antibody (Figures 4(a) and 4(b)). We established that the effect of neutralizing of CD14 exerted on sLPS binding in RAW264 cells was comparable to that of 50 *μ*g/mL of dextran sulfate, a competitive ligand of class A SR [34] which mediated uptake and detoxification of large quantities of LPS. Dextran sulfate at 50 *μ*g/mL inhibited sLPS-biotin binding by about 80%, whereas simultaneous action of 5 *μ*g/mL of the anti-CD14 antibody and 50 *μ*g/mL of dextran sulfate abolished the binding (Figures 4(a) and 4(b)). Both the anti-CD14 antibody and dextran sulfate also inhibited to a similar extent, by over 60%, the internalization of 1000 ng/mL of sLPS-biotin. A joint influence of these two agents very strongly diminished sLPS uptake, suggesting partial separation of CD14- and SR-dependent internalization routes of LPS (Figures 4(c) and 4(d)). Taken together, the data suggest that CD14 and SR participate in the binding and internalization of large quantities of sLPS while the CD14-mediated sLPS binding contributes only partially to TLR4 signaling that leads to TNF-*α* generation.

**Figure 5:**
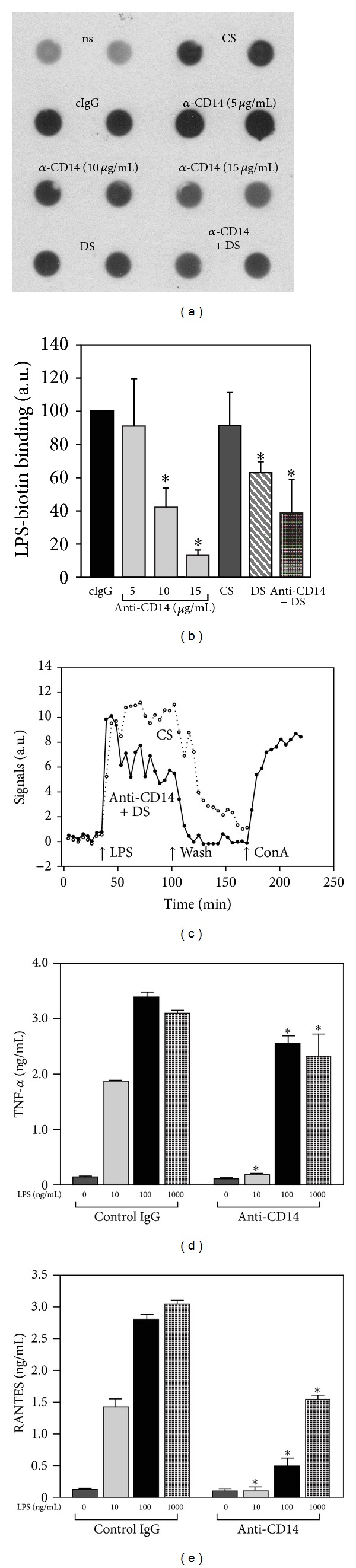
Disparate requirements for CD14 participation in the binding of high doses of LPS and production of TNF-*α* in J774 cells.(a, b) Binding of sLPS-biotin to the surface J774 cells. Cells were preincubated with 0.05% NaN_3_ (30 min, 37°C) and either left untreated (ns) or supplemented with 5, 10, or 15 *μ*g/mL of the anti-CD14 antibody or 10 *μ*g/mL isotype matched control IgG (cIgG) or 50 *μ*g/mL dextran sulfate (DS) or 50 *μ*g/mL chondroitin sulfate (CS) or a mixture of 5 *μ*g/mL anti-CD14 antibody and 50 *μ*g/mL dextran sulfate for 30 min (37°C). Subsequently, 1 *μ*g/mL of sLPS-biotin was added to the cultures for 1 h in the presence of 0.05% NaN_3_. (a) Dot-blot analysis of sLPS-biotin in cell homogenates. (b) Densitometric analysis of dot-blots exemplified in (a). Data are mean ± SEM from three experiments. (c) LigandTracer real-time analysis of the binding and internalization of sLPS-AF488. Cells were preincubated for 15 min at 37°C with a mixture of 10 *μ*g/mL anti-CD14 antibody and 50 *μ*g/mL dextran sulfate (DS) or with 50 *μ*g/mL chondroitin sulfate (CS) and transferred into the LigandTracer. After 30 min of the baseline setting (37°C), cells were exposed to 3 *μ*g/mL of sLPS-AF488 for 1 h (association phase), washed to remove the unbound LPS, and monitored for another 1 h to measure retention of sLPS-AF488 in cells. Traces with open symbols and closed symbols reflect sLPS-AF488 binding and internalization in CS-treated and anti-CD14/DS-treated cells, respectively. Concanavalin A-FITC (100 *μ*g/mL; ConA) was added to the anti-CD14/DS-treated culture for 40 min to ensure that the cells were still adherent. (d, e) Production of TNF-*α* (d) and RANTES (e) in cells pretreated with 10 *μ*g/mL of the anti-CD14 antibody or isotype-matched control rat IgG2b and stimulated with 10–1000 ng/mL of sLPS. Data are mean ± SEM from three experiments. *Significantly different from cells exposed to control IgG.

The data were reinforced by an analysis of participation of CD14 in sLPS binding and TNF-*α* production in J774 cells which express higher amounts of CD14 on the surface than RAW264 cells (not shown). The application of J774 cells allowed us to asses the amounts of sLPS-biotin bound to the surface of NaN_3_-treated cells after 1 h of incubation (Figures 5(a) and 5(b)). In addition, we were also able to perform a real-time analysis of the binding and internalization of sLPS-AF488 in the LigandTracer Green instrument which requires cells well adhering to the substratum (Figure 5(c)). The dot-blot analysis of the binding of 1000 ng/mL of sLPS-biotin revealed a dose-dependent inhibition of the binding by the anti-CD14 antibody. The binding was unaffected by 5 *μ*g/mL of the neutralizing antibody; however, it was reduced by about 58% at 10 *μ*g/mL and by about 77% at 15 *μ*g/mL of the antibody (Figures 5(a) and 5(b)). For comparison, 50 *μ*g/mL dextran sulfate inhibited the sLPS binding by J774 cells by about 40% but joint action of 5 *μ*g/mL of the anti-CD14 and 50 *μ*g/mL of dextran sulfate reduced the binding further by 61% (Figures 5(a) and 5(b)) pointing to the involvement of both CD14 and SR in the binding of high amounts of sLPS to cells. A control polyanion chondroitin sulfate at 50 *μ*g/mL did not affect the binding or internalization of sLPS in RAW264 and J774 cells (Figures 4, 5(a), and 5(b)).

These data on CD14 and SR engagement in sLPS association with cells were confirmed by an analysis of a real-time binding and internalization of sLPS-AF488 to living J774 cells performed in the LigandTracer instrument. For this analysis, J774 cells were pretreated with 10 *μ*g/mL of the anti-CD14 antibody and 50 *μ*g/mL of dextran sulfate or with 50 *μ*g/mL of chondroitin sulfate in a control. Subsequently, the cells were placed into the LigandTracer at 37°C and exposed to 3 *μ*g/mL of sLPS-AF488. In these conditions, an association of sLPS-AF488 with cells (including LPS binding and internalization) was monitored for 1 h, after which the excess of sLPS-AF488 was washed out and the retention of endotoxin in cells was assessed for another 1 h. As can be seen in Figure 5(c), simultaneous exposition of cells to dextran sulfate and the CD14-neutralizing antibody suppressed both the association and retention of 3 *μ*g/mL sLPS-AF488 in cells, apparently abolishing its accumulation in cells. The lack of retention of sLPS-AF488 was not caused by a detachment of cells from the substratum, since they were still able to bind FITC-labeled concanavalin A (Figure 5(c)). Taken together, the data indicate that CD14 and SR mediate the binding and internalization of large quantities of LPS. Despite the participation of CD14 in the binding of high amounts of sLPS, neutralizing of CD14 with 10 *μ*g/mL of the 4C1 antibody in J774 cells inhibited TNF-*α* production induced by 100 or 1000 ng/mL of sLPS by 27–35% only. The function blocking anti-CD14 antibody exerted, however, strong inhibitory effect on TNF-*α* released by J774 cells at 10 ng/mL of sLPS, consistent with the results obtained in RAW264 cells (Figure 5(d) compared with Figure 1(a)). RANTES production in J774 cells induced by sLPS was markedly inhibited by the anti-CD14 antibody (Figure 5(e)). Taken together, the data suggest that the interference with the binding of high amounts of sLPS to CD14 has more pronounced inhibitory effect on the association of LPS with cells than on TNF-*α* production.

### 3.3. Modulation of TNF-*α* Production by SR

**Figure 6:**
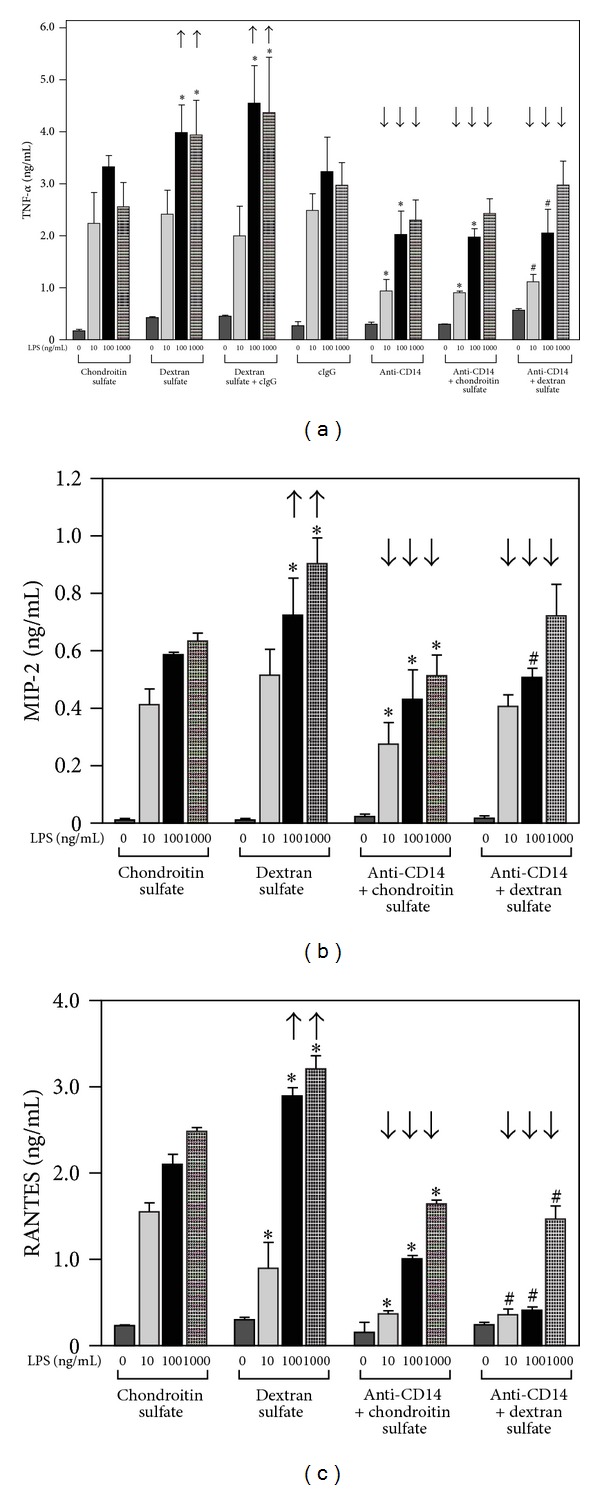
Occupation of SR by dextran sulfate upregulates sLPS-induced TNF-*α* production with CD14 participation. RAW264 cells were pretreated with 50 *μ*g/mL dextran sulfate or 50 *μ*g/mL chondroitin sulfate or 10 *μ*g/mL anti-CD14 or 10 *μ*g/mL isotype-matched rat IgG2b (cIgG) or combination of the agents, as indicated, and subsequently were exposed to 10–1000 ng/mL of sLPS (37°C). Generation of TNF-*α* (a), MIP-2 (b), and RANTES (c) was estimated in culture supernatants by ELISA tests. Arrows directed upwards point to the enhancement of cytokine production stimulated by 100 or 1000 ng/mL of sLPS in the presence of dextran sulfate while arrows directed downwards indicate the inhibition of cytokine generation by the anti-CD14 in comparison to cells exposed to sLPS accompanied by dextran sulfate. Data shown are mean ± SEM from three or four experiments each run in triplicates. *Significantly different from cells exposed to chondroitin sulfate and sLPS; ^#^significantly different from cells exposed to dextran sulfate and sLPS.

The interference with the LPS/CD14 interaction by the 4C1 antibody only partially reduced TNF-*α* production induced by high doses of LPS (100–1000 ng/mL) which suggested the involvement of other LPS acceptors, like SR, in this process. To test this assumption we measured TNF-*α* production in RAW264 cells stimulated with 10–1000 ng/mL of sLPS in the presence of dextran sulfate. An attenuation of the cytokine release in these conditions would indicate the importance of LPS/SR interaction for TNF-*α* production. However, blocking of sLPS binding to SR by dextran sulfate was found to upregulate rather than inhibit TNF-*α* production induced by 100 or 1000 ng/mL of sLPS. In these conditions, 50 *μ*g/mL dextran sulfate either alone or in combination with a control antibody enhanced TNF-*α* release by 20–70% (Figure 6(a)). Similar enhancement was found for MIP-2, another cytokine produced mainly in MyD88-dependent manner (Figure 6(b)), and for RANTES generated in MyD88-independent manner (Figure 6(c)). The presence of dextran sulfate did not affect significantly production of TNF-*α* and MIP-2 induced by 10 ng/mL of sLPS while RANTES production was moderately inhibited in these conditions (Figures 6(a)–6(c)). Of note, the stimulatory effect exerted on TNF-*α*, MIP-2, and RANTES production by dextran sulfate at 100–1000 ng/mL LPS was reversed by a simultaneous interference with the LPS/CD14 interaction by the 4C1 antibody (Figures 6(a)–6(c)). In these conditions, the production of TNF-*α* reached the level found in cells exposed to the anti-CD14 antibody alone (Figure 6(a)). These data suggest that the binding of LPS to CD14 is required to support the enhanced TNF-*α* production stimulated in the presence of dextran sulfate.

## 4. Discussion

TNF-*α* is a major pro-inflammatory cytokine produced by mammals during infection with Gram-negative bacteria. This cytokine is generated mainly by macrophages which reside in many tissues and trigger an array of innate immune responses upon encounter of invading pathogens [35]. Monocyte-to-macrophage differentiation is accompanied by upregulation of CD14 expression [36]. Therefore, macrophages are prone to recognize LPS monomers (provided by LBP) by the binding of the lipid A part of LPS molecule to the hydrophobic pocket of CD14 from which endotoxin can be transferred to the TLR4/MD-2 [11, 12, 37]. A line of studies performed on living cells indicates that LPS induces formation of multimolecular complexes in the plasma membrane composed of TLR4/MD-2, CD14, and several other proteins potentially involved in LPS recognition [23–25]. To asses participation of CD14 in signaling pathways leading to LPS-induced TNF-*α* production we conducted studies on RAW264 and J774 macrophage-like cells. Our preliminary data indicated that these cells differed greatly in the level of expression of CD14 on their surface; cytometric analysis revealed that the fluorescent signal attributed to CD14 was 2.6-fold stronger in J774 cells than in RAW264 cells (not shown). In both cell lines CD14 was found indispensable for TNF-*α* production induced by 1 ng/mL of sLPS or rLPS. However, TNF-*α* production induced by higher concentrations of sLPS and rLPS (100 or 1000 ng/mL) required CD14 to a much lower extent. The inhibition of TNF-*α* generation exerted by the CD14 neutralizing antibody reached 25–27% at 1000 ng/mL of sLPS in RAW264 and J774 cells. These data indicate that at higher LPS concentrations participation of CD14 in TNF-*α* production can be partially omitted. On the other hand, a reduction of TLR4 level in cells by half correlated with 50–60% inhibition of TNF-*α* and RANTES production induced by 1–1000 ng/mL of sLPS and rLPS. This indicates that the cytokines were generated in TLR4-dependent manner, although further confirmation of these results will require studies on TLR4^−/−^ macrophages. Our data are in line with results obtained on human monocytes exposed to the function blocking anti-CD14 antibody MY4 and bone marrow-derived dendritic cells or macrophages of CD14^−/−^ mice stimulated with rLPS or sLPS, respectively [18, 25]. It seems likely that, at high LPS doses, endotoxin molecules can bind either directly to TLR4/MD-2 complex or be transferred to the signaling complex by other LPS-binding proteins localized in the plasma membrane proteins [24]. It was reported recently that albumin also forms complexes with LPS monomers and can transfer endotoxin directly to MD-2 protein [38].

The interference with LPS/CD14 interaction by the anti-CD14 antibody inhibited rLPS-induced TNF-*α* production relatively weaker in comparison to TNF-*α* generation induced by sLPS (Figure 1; compare (a) and (c)). In addition, both LPS forms displayed a striking difference in the FBS requirement for the initiation of TNF-*α* production, and lack of which nullified sLPS activity. These data indicate the differences between sLPS and rLPS modes of action which are just being appreciated. In 2005 Beutler's group showed that rLPS activity is less CD14-dependent compared to sLPS to induce TNF-*α* production in murine peritoneal macrophages, and recently these observations were reinforced by studies of sLPS- and rLPS-induced TNF-*α* production in murine dendritic cells [9, 39]. Disparate requirements for participation of CD14 and FBS (as LBP source) for sLPS- or rLPS-induced IL-6 production were found also in murine mast cell lacking CD14 expression [10] and more recently for TNF-*α* production in human macrophages [40]. These different requirements of sLPS and rLPS for accessory proteins to mediate cell activation can be attributed to differences in physicochemical properties between these two forms of LPS. It was suggested that highly hydrophobic rLPS can incorporate directly into the plasma membrane to get an access to TLR4 [10]. Furthermore, it was shown that rLPS aggregates rather than monomers are the biologically active form of endotoxin [41] which could explain why rLPS can act without participation of LPS monomer-binding proteins. It is tempting to speculate that aggregates of rLPS (100–1000 ng/mL) can be also responsible for the CD14- and FBS-independent production of RANTES in RAW264 cells (Figure 1(d)). Recently, Watanabe et al. demonstrated that LPS-liposomes can induce RANTES generation without CD14 participation [42]. Otherwise, CD14 is required for sLPS-induced endocytosis of activated TLR4 followed by TRIF-dependent synthesis of cytokines like RANTES ([18]; see also Figure 1(c) in this paper).

In contrast to the conditional involvement of CD14 in sLPS-induced cytokine production, CD14 seems to be crucial for the binding of high amounts of sLPS to the surface of RAW264 and J774 cells. After the blocking of the function of CD14 with 10 *μ*g/mL of the anti-CD14 antibody in RAW264 cells, the binding and internalization of 1000 ng/mL sLPS-biotin were reduced by about 90% and 64%, respectively, while TNF-*α* production induced by 1000 ng/mL of sLPS was diminished by 25% only (compare Figures 4(b), 4(d), and 1(a)). Similar discrepancy in the magnitude of inhibition of sLPS binding and TNF-*α* production was found in J774 cells exposed to 10 *μ*g/mL of the anti-CD14 antibody (Figures 5(b) and 5(d)). More pronounced sensitivity of sLPS binding to the inhibition by the anti-CD14 antibody in RAW264 cells than in J774 can be attributed to the differences in CD14 surface amounts between these cells. Taken together, the data indicate that in macrophages CD14 participates in the binding and internalization of large quantities of sLPS, similarly as indicated for monocytes [21, 43]. However, CD14-mediated binding and internalization of sLPS are required only to a certain extent for the signaling of TLR4 and TNF-*α* production. The internalized sLPS serves possibly as an activator of TRIF-dependent signaling pathway of TLR4 in endosomes or is directed for detoxification. The participation of CD14 in the internalization of high doses of sLPS was reflected by sustained reduction of the cell surface level of CD14, reaching 21% after 1 h and 38% after 2 h of J774 cell stimulation with 1000 ng/mL of sLPS (not shown).

Significant portion of sLPS can be bound and internalized also with the participation of SR judging from the attenuation of those processes by dextran sulfate, a competitive ligand of SR. Among scavenger receptors, SR-A was indicated earlier as mediating uptake of large quantities of LPS in macrophages having a protective function against excessive pro-inflammatory responses to LPS [19, 20, 44]. Participation of SR in removal of the excess of sLPS can explain why TNF-*α*, MIP-2, and RANTES production increased in conditions when SR/LPS binding was inhibited by dextran sulfate (Figure 6). Recent data indicate, however, more complex scenario of SR-A involvement in LPS-induced cytokine production [22, 45]. As we reported earlier, activation of SR-A by dextran sulfate can upregulate CD14 and TLR4 expression on the cell surface and contribute this way to the high TNF-*α* production when both LPS and dextran sulfate are present [22]. The function blocking anti-CD14 antibody reversed the enhancement of TNF-*α* generation induced by 100–1000 ng/mL sLPS accompanied by dextran sulfate (Figure 6) indicating that participation of CD14 is a limiting factor in this SR-related activity. It should be noted that both CD14 and SR mediate the binding of high doses of sLPS since this process was strongly inhibited by the anti-CD14 antibody and dextran sulfate (Figures 4 and 5). Although an additive effect of these two agents suggests a partial separation of CD14- and SR-dependent binding of sLPS, a cooperation between these two receptors is possible. Taking into account that CD14 is a plasma membrane raft protein [46] and SR-A associates with caveolae/rafts [47] these regions of the plasma membrane can serve as platforms for the putative interaction between CD14 and SR-A. This suggestion is supported by recent report demonstrating that activation of SR-A enhanced the interaction of SR-A, caveolin, and major vault protein (MVP) located in rafts which led to the increase of TNF-*α* production in RAW264 cells [48].

## 5. Conclusion

Our data suggest that CD14 participates in the binding and internalization of large quantities of sLPS but these events only to some extent are required for signaling leading to TNF-*α* production. The generation of TNF-*α* induced by rLPS is even less dependent on CD14. Besides CD14, SR are involved in the binding of large quantities of sLPS and the involvement of SR modulates sLPS-induced TNF-*α* production.
